# Phrenology—Physiognomy

**Published:** 1826-10-01

**Authors:** 


I. *A System of Phrenology*. By George Combe, President of the Phrenological Society, &c. &c. 2nd Edition, 8vo. Edinburgh and London, 1825, pp. xxiv. 566.

II. *Phrenology, in connexion with the Study of Physiognomy*. By G. Spurzheim, M.D. Part I. Characters, with 34 Plates. Treuttel and Wurtz, London, July, 1826.

Phrenology is more intimately connected with the applications of medical knowledge than may at first sight be apparent: on this account, therefore, we recognize in the science of its principles a legitimate and useful subject of professional inquiry. We must acknowledge, at the same time, that we feel impelled, by the pure force of multifarious and unquestionable evidence, to regard this as the most intelligible and self-consistent system of mental philosophy that has ever yet been presented to the contemplation of inquisitive men. Similar sentiments, we doubt not, will be excited in the mind of every one who impartially examines this very comprehensive work of Mr. Combe's; and, to such of our readers as may not be intimately acquainted with the sublimer doctrines of his favourite science, we hope to do an essential service in affording them an opportunity of retracing the method, at once profound and simple, by which this excellent moralist unfolds the more manifest as well as the most mysterious "bearings" of our nature. In attempting this important duty, however, we shall be obliged, for obvious reasons, to pass, with a reference only, the questions in phrenology, which we had illustrated at considerable length, in our Numbers for March, 1823, and March, 1824; but, with regard to these and to the views of Mr. Combe, which we now propose to exhibit in a state of analytical concentration, we ask nothing else than their being calmly investigated by the laws of inductive philosophy. Let none of us admit any doctrine of phrenology, because its advocates affirm its truth: neither let us, on the other hand, despise or condemn it merely because this has been done (in too many instances without examination) by individuals, in courteous phrase, denominated wise men and philosophers. The phrenologists may err, and their adversaries may err, but Nature is ever true in all her manifestations; let us, therefore, observe and judge of these for ourselves, without being prejudiced by the tiny pratings of vain and ignorant persons who affect to depreciate the phrenological system without so much as understanding the elementary principles on which it is constructed.

Mr. Combe opens his work with some introductory observations which include questions, in the highest degree important; they are stated with great perspicuity; in general, they are profound and eloquent: his reasonings, in our mind, are quite demonstrative of his doctrines. He defines phrenology to be a system of philosophy of the human mind adapted to explain the primitive *powers* of feeling which incite mankind to action, and the *capacities* of thinking that guide our actions till we attain the object of our desires. In proceeding to take a view of the mental manifestations, he commences with a remark which is too concise to admit of analysis, and too exquisite to be otherwise than debased by change of diction: we would, therefore, retain its original beauty in an entire transcription.

"The human mind," he observes, "as it exists in this world, cannot, *by itself*, become an object of philosophical investigation. Placed in a material world, it cannot act, or be acted upon, but through the medium of an organic apparatus. The soul, sparkling in the eye of beauty, does not transmit its sweet influences to a kindred spirit, but through the filaments of an optic nerve; and even the bursts of eloquence which flow from the lips of the impassioned orator, when mind appears to transfuse itself almost directly into mind, emanate from, and are transmitted to, corporeal beings through a voluminous apparatus of organs. If we trace the mind's progress from the cradle to the grave, every appearance which it presents, reminds us of this important truth. In earliest life, the mental powers are feeble as the body, but when manhood comes, they glow with energy, and expand with power; till, at last, the chill of age makes the limbs totter, and the fancy's fires decay. Nay, not only in the great stages of our infancy, vigour and decline, but the experience of every hour reminds us of our alliance with the dust. The lowering clouds and stormy sky depress the spirits and enerve the mind;—after short and stated intervals of toil, our wearied faculties demand repose in sleep;—famine or disease is capable of levelling the proudest energies in the earth;—and even the finest portion of our compound being, the mind itself, *apparently* becomes diseased, and leaving Nature's course, flies to self-destruction to escape from pain. These phenomena must, however, be referred to the organs with which, in this life, the mind is connected; but, if the organs exert so great an effect over the mental manifestations, no system of philosophy is entitled to consideration, which would neglect their influence, and treat the thinking principle as a disembodied spirit. The phrenologist, therefore, regards man as he exists in this sublunary world; and desires to investigate the laws which regulate the connexions between the organs and the mind, but without attempting to discover the *essence* of either, or the *manner* in which they are united."

Our author next adverts to the prejudice which has arisen against the physiology of man from the defective practice of Locke, Hume, Reid, Stewart, Brown, and other distinguished metaphysicians, in studying the mind with too little reference to the body; as if, he says, the mind were degraded by contemplating it in connexion with matter: but man, he adds, is the work of the Creator of the universe, and no part of his constitution can be unworthy of regard and admiration. The whole phenomena of life, Mr. C. very justly observes, are the result of mind and body joined, each modifying each; and how, he asks, can we explain a result, without attending to *all* the causes which unite towards its production? Equally erroneous as this, is the false and vulgar notion of those persons, who would teach us that the mind is nought but a combination of matter, and that its functions may be explained by mechanical motions in its parts: the only legitimate and philosophical method of investigating the question, therefore, consists in *observing* the laws that regulate the union of the corporeal and mental parts of man, without pretending to discover the essence or *modus operandi* of either.

Mr. C. then directs our attention to the variety of the mind's endowments, and to the remarkable circumstance of "the savage and the sage" having in all ages admitted it to be possessed of different powers; but, at the same time, being utterly at variance with regard to the number and nature of the mental faculties. This leads him to expose the discordant hypotheses advanced on this head by the ontological sophists of all kinds, from Aristotle to the late amiable and erudite Dr. Brown, who have puzzled themselves and the readers of their books with attempts at the mysterizing of subtilties, of which their own fancies were the principal source. He concludes, by shewing that, after the lapse and labour of more than two thousand years, philosophers are not yet agreed concerning the existence of many of the most important principles of action, and intellectual powers of man. How can they be so agreed, since they have all along conducted their speculative inquiries on foundations manifestly unphilosophical and by a method as manifestly imperfect? Ten men, we might say, or twenty men, or ten times twenty men, may pass all the days of a lengthened age in ruminating, each on the modifications of his own consciousness; but how, we might then ask, can their observations, so few in number and so circumscribed in sphere, be capable of explaining even a tittle of the infinitude of mental manifestations exhibited by the myriads of mankind under the countless influences of constitution and circumstances, to which all their natures are perpetually exposed? The thing is obviously and altogether impossible. When we find two human countenances in all respects identical, then, but not till then, may we hope to discover two identically constituted minds. If, therefore, says Mr. C. phrenology could introduce into the philosophy of mind even a portion of the certainty and precision which attend physical investigations, it would confer no small benefit on this interesting department of science; and this much, notwithstanding the noisy opposition it has sustained, we believe it to be fully competent to do, because its principles are uniform as the elements of nature, and permanent as the eternity of truth.

Supposing the number and nature of the primitive mental faculties to be ascertained, it is to be observed, Mr. C. adds, that in actual life they are successively developed. The infant feels before it is observant; it observes occurrences long before it reasons: and it feels love, fear, attachment, before it is alive to the sublime or the beautiful: hence, a correct theory of mind ought to unfold principles to which these facts may be referred. Even after the maturity of age, how *different are the degrees* in which we are endowed with the various mental powers! Admitting, he goes on to say, each individual to possess all the faculties, the assemblage of which constitutes the human mind, in what a *variety of degrees of relative strength* do they appear in *different* persons? We quote his illustrations, admiring their conclusiveness and impressive eloquence.

"In one," he says, p. vi. "the love of glory is the feeling which surpasses all; another is deaf to the voice of censure, and callous to the accents of applause. The soul of one melts with softest pity at a tale of woe; while the eye of another never sheds a sympathetic tear. One individual spends his life in an ardent chace of wealth, which he stops not to enjoy; another scatters in wasteful prodigality the substance of his sires, and perishes for want, from a mere incapacity to retain. One vast intellect, like Newton's, fathoms the profundities of science; while another feeble mind scarcely gropes its way through the daily occurrences of life. The towering imagination of a Shakespeare, or a Milton, soars beyond the boundaries of sublunary space; while the sterile fancy of another sees no glory in the Heavens, and no loveliness on earth. A system of mental philosophy, therefore, pretending to the truth of nature, ought not only to reveal the simple elements of feeling and of thought, but enable us, likewise, to discover in what *proportions* they are combined in different individuals. In chemistry, one combination of elementary ingredients produces a medicine of sovereign virtue in removing pain; another combination of the same materials, but differing in their relative proportions, brings forth a mortal poison: so, in human nature, one combination of faculties may produce the midnight murderer and thief; and another, a Franklin, a Howard, or a Fry, glowing with charity to man."

Instead, however, as our author well observes, of obtaining rules by which to discriminate the effects produced upon the character and conduct of individuals by different combinations of the mental powers, we find the works of philosophers on the mind to be only a never-ending series of disputes, whether such differences do exist in nature or are the result of education and other adventitious circumstances. Hence, this department of the science of man remains a perfect waste, uncultivated and unimproved; and hence, on surveying it as exhibited in these recondite discussions, we perceive, says Mr. C. *first*, That no account is given of the influences of the material organs on the *manifestations* of the mental powers; and that the progress of the mind from youth to age, and the phenomena of sleep, dreaming, idiotcy and insanity, are left unexplained or unaccounted for, by any principles admitted in their systems; *secondly*, That the existence and functions of some of the most important primitive faculties are still in dispute; and, *thirdly*, That no light whatever has been thrown on the nature and effects of combinations of the primitive powers in different degrees of relative proportion.

From this Mr. Combe goes on to consider the investigations of human nature by another class of observers,—moralists, poets, and divines. His remarks on this head are most pertinent.

"These," he says, p. ix. "have looked upon the page of life merely to observe the characters there exhibited, with the view of tracing them anew in their compositions: and certainly they have executed their design with great felicity and truth. In the pages of SHAKESPEARE, ADDISON, JOHNSON, TILLOTSON, and BLAIR, we have the lineaments of mind traced with a perfect tact, and exhibited with matchless beauty and effect. But these authors had no systematic object in view, and did not aim at connecting their observations by principles which might render them subservient to the *elucidation* of the phenomena of life, in less skilful hands than their own. Hence, although in their compositions we find ample and admirable materials for the elucidation of a true system of the philosophy of man, yet, without other aids than they supply, we cannot arrive at the fundamental principles which ought at once to unite and explain their observations. Phrenology, therefore, if a true system of human nature, ought to furnish, to the popular reader, the key of philosophy to unlock the stores of intellectual wealth contained in the volumes of our most admired and instructive authors."

Mr. C. next surveys the processes by which the physiologists have endeavoured to ascertain and determine the parts of the body with which the several mental powers are most closely connected. Some of them have dissected the brain, in the hope of discovering in its texture an indication of the functions which it performs in relation to the mind: when we examine, however, with the most scrupulous minuteness, the form, colour, and texture of the brain, no sentiment can be perceived slumbering in its fibres; no half-formed ideas starting from its folds: it appears to the eye only as a mass of curiously convoluted matter, and the understanding declares its incapacity to penetrate the purposes of its parts. The moral sentiments have been derived, by others, from various viscera, or from the plexus and ganglia of the great sympathetic nerve; but comparative anatomy and physiology entirely contradict this opinion. There are animals endowed with faculties attributed to certain viscera, which do not possess these viscera: insects, for instance, become angry, and have neither liver nor bile. Oxen, horses, and even hogs, have many viscera in structure analogous to those of man, and yet they want many faculties which are ascribed to these viscera, and with which man is endowed. The heart has been regarded as the seat of the tender affections; but the heart of the tiger and of the lamb are alike in structure, and the one ought to be the organ of cruelty and the other of meekness, if this supposition were true. Others, again, have compared the size of the brain of man with that of the lower animals, contrasting, at the same time, their mental powers; and have been led to conclude that it is the organ of mind, and that its greater development in man indicates his mental superiority over the brutes: but, says Mr. C. these philosophers have not succeeded in determining the functions of the different parts of this organ, and have not in any important degree been able to connect their discoveries with the philosophy of mind. Camper, in order to measure the brain's dimensions, and, as he imagined, the corresponding energy of the mental powers, adopted the *facial angle*; but, as this angle applies only to the anterior parts of the brain situated in the fore-head, to the exclusion of all the lateral and posterior parts; and as, in many negroes, the jaw-bones are extremely prominent and the facial angle acute, while their foreheads are in fact largely developed and their intellectual faculties powerful, yet, by this rule, they ought to be inferior to many stupid Europeans, whose foreheads are deficient and whose jaws recede. Hence, the facial angle cannot serve as a means of measuring the moral sentiments and intellectual faculties. According to SOEMMERRING and CUVIER, who compare the size of the brain in general with that of the face, animals are more stupid as the face is larger in proportion to the brain. The infallibility of this rule, however, is easily proved; because LEO X. MONTAIGNE, LEIBNITZ, HALLER, and MIRABEAU, had large faces and very considerable brains; BOSSUET, VOLTAIRE, and KANT had, on the contrary, small faces and also large brains. The cerebral parts, in fine, have likewise been compared with each other, as the brain with the cerebellum, with the medulla oblongata, or with the nerves, in order to ascertain their functions; but these modes, like the others, have led to no satisfactory results. We all know, indeed, that no theory of the functions of the brain is yet admitted and taught in the schools as certain science, such as the doctrine of the circulation of the blood and the offices of the muscles and nerves.

With these observations, the importance of which we are desirous of impressing on the minds of our readers, Mr. Combe naturally arrives at the inquiry, "in what manner, then, do the phrenologists pretend to succeed in an investigation which has baffled so many ingenious men?" This leads him to give an historical account of the origin and progressive development of the new philosophy; but, having retraced this in a former article, we shall conclude this branch of our subject with the observations of an illustrious metaphysician which, says Mr. C. are peculiarly applicable to the history and prospects of phrenology. "TRUTH" (it is Locke who speaks,) "*scarce ever yet carried it by vote any where, at its first appearance.* NEW OPINIONS *are always suspected, and usually opposed without any other reason, than because they are not common. But* TRUTH, *like gold, is not the less so, for being newly brought out of the mine. 'Tis trial and examination must give it price, and not any antique fashion; and, though it be not yet current by the public stamp, yet it may, for all that, be as old as nature, and is certainly not the less genuine.*" If these excellent sentiments exert the right influence on the mind of an unprejudiced medical inquirer after truth, they will naturally affect it with intermingled feelings of shame and exultation, in reverting to the persecutions of our immortal HARVEY!—this true philosopher was long and loudly reviled as *a knave and a quack*, for discovering and teaching the circulation of the blood!

By this sketch of his introductory observations now submitted to their attention, our readers will be satisfied, without further evidence, that Mr. Combe is a logician of no ordinary powers, and that his manner of philosophizing is as temperate and dignified as his arguments are conclusive. The pure desire of imparting useful knowledge and of establishing instructive truth, exclusively of all other ends, is so remarkable and manifest in each of his descriptions, discussions, and inductions, that every upright inquirer, whatever may be his sentiments regarding the true value of phrenology, must approve and even be delighted with the generous zeal and philanthropy which universally pervade our author's "System," and render it a beautiful model of scientific doctrine. For this reason we are solicitous of persuading all those, whom our own example may tend to influence, to examine it with deep attention; and, on their doing this, we can assure them that, though it should fail of inducing them to admit the particular views of Mr. C. it will at least convince them of its being powerfully adapted to make us good men and liberal philosophers.—As this valuable work itself is, in fact, an analysis of an intricate and comprehensive science, and by consequence in a great measure insusceptible of condensation, we shall premise a synoptical outline of its subjects and then make such selections, in extract and abridgement, as may conduce to the promotion of our object, on this and every other occasion;—viz. *a spirit of free and rational inquiry into the nature and applications of* TRUTH.

Mr. Combe distributes his "System" into these four principal divisions;—principles of phrenology; division of the faculties; modes of activity of the faculties; objections to phrenology, and an appendix, containing arrangements of the organs by Drs. Gall and Spurzheim; notes on individuality by Mr. Scott and the Rev. Mr. Welsh, and an interesting addition to the section on the harmony of the faculties from the author's own pen.

I. PRINCIPLES OF PHRENOLOGY.—Two important propositions are explained and demonstrated, at the outset of this division, viz. That the dispositions and capacities of individuals can be discovered; and that the size of different parts of the brain can be ascertained, during life. The chief difficulties in discovering the form of the brain are next considered, and satisfactorily removed. Mr. C. then proceeds to teach the practical application of the phrenological principles; and, under this head, he inculcates the fundamental doctrine,—*that* SIZE *in the cerebral organs indicates* POWER *in the mental faculties*; shews with much precision, that *power* is distinguishable from *activity*; and gives rules how to estimate size as well as to designate its relations in terms and numerals. This is followed by some instructive remarks on the brains of the lower animals, and on the circumstances which modify the effects of size: this part of the work concludes with a definition of *activity*, which means the *rapidity* wherewith the faculties may be manifested; and with illustrations of the aphorism, *that the* LARGEST *organs in each head have the* GREATEST, *and the* SMALLEST *the* LEAST, *tendency to natural activity*.—We cannot pass from this section without entreating our readers, as they value the improvement of *medical* philosophy, to examine these questions dispassionately: an extract from the "System" may serve as a foretaste of the satisfaction such an inquiry will afford to the unprejudiced mind.

"Human phrenology," says Mr. C. p. 46, "is founded, not on analogy, but on positive observation. Some persons are pleased to affirm, that the brains of the lower animals *consist of the same parts* as the human brain, only on a *smaller scale*: but this is highly erroneous. If the student will procure brains of the sheep, dog, fox, calf, horse or hog, and compare them with the human brain, he will find a variety of parts, especially in the convolutions which form the organs of the moral sentiments and the reflecting faculties, *wanting* in these animals."—"It is proper to advert to certain conditions which may co-exist in the brain with *size*, and to attend to their effects. *Power* in the manifestations and *size* in the organ, are in the *general case* proportionate; and when differences in size are considerable, no circumstance, consistent with health, will render the manifestations equal in power: one brain, however, may be more perfect in constitution than another, and in consequence act more vigorously, although not larger in dimensions; but these differences are slight and their effects limited. Size, then, is not the *only* requisite to the manifestation of great mental power: the brain must possess also a healthy constitution and that degree of *activity* which is the usual accompaniment of health. Now, the brain, like other parts of the body, may be affected with certain diseases, which do not diminish or increase its magnitude, and yet *impair its functions*; and, in such cases, great size may be present, and very imperfect manifestations appear: or, it may be attacked with other diseases, such as inflammation, or any of those particular affections *whose nature is unknown*, but to which the name of *mania* is given in nosology, and which greatly exalt its action; and then very forcible manifestations may proceed from a brain comparatively small: but it is not less true, that when a larger brain is excited to *the same degree by the same causes*, the manifestations become increased in *energy* in proportion to the increase of *size*. These cases, therefore, form no valid objection to phrenology: the phrenologist ascertains, by previous inquiry, that the brain is in a state of health; if it is not, he makes the necessary limitations in drawing his conclusions."—"Nature admits of no exceptions, and a single instance of decidedly vigorous manifestations, with a small organ, disease being absent, would overturn all previous observations in favour of that organ: but men are liable to err; and, although an individual phrenologist may have called an organ small, the manifestations of which are powerful, or *vice versâ*, this is not to be precipitately charged against Nature as an exception. Chemists occasionally fail in experiments, mathematicians err in demonstrations, and arithmeticians are wrong in calculations; and, in like manner, phrenologists may commit mistakes in observing cerebral development. The test in such cases is, to compare the organ in regard to which an apparent discrepancy has occurred, with the same organ in the head of a person whose powers of manifestation are KNOWN to be diametrically opposite; and, if the organs are not perceived by an ordinary eye to differ, then the exception is proved."—"If, in each of two individuals the organs of propensity, sentiment, and intellect, are equally balanced, the general conduct of one may be vicious and that of another moral and religious. But the question here is not one of *power,* for as much *energy* may be displayed in vice as in virtue; but it is one of *direction* merely. Now, in cases where an equal development of *all* the organs exists, *direction* depends on *external* influences, and then no phrenologist pretends to tell to what objects the faculties have been directed, by merely observing the size of the organs."

II. DIVISION OF THE FACULTIES.—Mr. Combe, in his classification of the mental faculties, continues the arrangement which has been in use, in Britain, since 1815, not because he regards it as the most perfect, but on account of its being as yet the most convenient and familiar: he thinks it preferable, as phrenology is manifestly a progressive science, to delay proposing alterations till they can be made permanent. Looking at the innumerable facts presented by observation in proof of the doctrine, he concludes, that the mind consists of an aggregate of faculties or powers, and the brain of an aggregate of organs; that every *faculty* of the mind has its own determinate *organ* in the brain; and that, though the *essence* of the mind be altogether indefinable, its cerebral organs are material, visible, tangible, and readily distinguishable. At the same time, instead of overwhelming his readers with the illimitable mass of evidence on which phrenology is founded, he merely refers them to the various works in which the facts and cases are detailed: but above all things, he says, let those persons who desire philosophical conviction, resort directly to *Nature,* which is always within their reach; for SELF-CONVICTION CAN BE OBTAINED ONLY BY SELF-OBSERVATION.

Mr. C. distributes the whole mental system into two primary orders—*feelings and intellectual faculties*—which, with their subordinate divisions, we shall exhibit under a synoptical enumeration, interspersed with an extract or occasional remark.

ORDER I. FEELINGS: This contains two genera, the *propensities* and *sentiments.*

GENUS I. PROPENSITIES.—These faculties do not form ideas, and are common to man with the lower animals: their sole function is to produce a propensity or instinctive impulse of a specific kind. Nine of these are established by all kinds of evidence: they have been denominated, *amativeness, philoprogenitiveness, concentrativeness, adhesiveness, combativeness, destructiveness, constructiveness, acquisitiveness,* and *secretiveness.* At p. 113 of the "System," is a very interesting sketch on the *appetite for food*, as a propensity, and the situation of its organ in the brain. Dr. Hoppé of Copenhagen, is of opinion, that, besides the nerves of the stomach and palate, an affection of which gives rise to the sensations of hunger and thirst, there must also be an organ in the brains of animals for the instinct of *nutrition* (taking nourishment for the preservation of life,) which incites them to the sensual enjoyments of the palate, and the activity of which is *independent* of hunger and thirst. How, he asks, should the mere sense of hunger, more than any other disagreeable or painful sensation, make the animal desire food, the necessity of such not being known to him by experience? This could only be effected by *instinct*, because either an instinct, *i. e.* the immediate impulse of an organ, or else experience and reflection are the causes of all actions. From considerable observation, Dr. H. concludes *(Mr. Combe had previously arrived at a similar induction which, however, his modesty led him to regard as probable only)* that the "organ for taking nourishment" is situated in the zygomatic fossa, and that when fully developed, it occasions a remarkable largeness of the face or head, altogether distinct from that caused by prominent cheek-bones. We recommend this subject and the discussion of it, to those of our readers whose propensities impel them to desire the elucidation of philosophical truth.

Genus II. Sentiments.—The feelings produced by this kind of faculties are not the *immediate* consequences of the presence of external objects: they are excited, *only indirectly*, through the medium of intellectual perceptions or sensations. They differ from intellectual perceptions in being accompanied with a peculiar vividness, which every one understands, but which it is impossible to express by any verbal definition: they may, moreover, exist with great intensity, by the internal activity of their organs. These faculties have been named *sentiments*, because they determine a propensity to act, joined with an emotion or feeling of a particular kind: they correspond to the class of "*Emotions*" of the metaphysicians. Several of them are common to man and the inferior animals; others are peculiar to man: the former are styled the *lower*, the latter the *higher* sentiments.

1*st. Lower.*—These are four in number, and have been designated, *self-esteem, love of approbation, cautiousness, and benevolence.* In his illustrations of the sentiment of cautiousness, Mr. C. introduces a general remark which ought always to occupy the student's attention. It is a principle in phrenology, he says, that absence of one quality never confers another. Every feeling is something *positive* in itself, and is not a mere negative of a different emotion: fear, for instance, is a positive feeling, and not the mere want of courage; it is produced by the faculty of *cautiousness*, which tends to make the individual apprehend danger, and thus lead him to hesitate before he acts, and to trace consequences that he may be assured of his safety. Its full endowment is essential to a prudent character: it produces a cautious, circumspect, and considerate disposition of mind.

*2nd. Higher.*—Besides the faculties and their organs already defined as common to man with the brutes, he is endowed with a variety of sentiments, which constitute the human character, and of which the lower creatures are entirely destitute: the cerebral parts, moreover, that constitute the organs of these faculties are totally wanting in the brains of irrational animals. Such sentiments are named, *veneration*, *hope*, *ideality*, *conscientiousness*, and *firmness*. Additionally to these, is the sentiment of *wonder*, the organ of which is now ascertained to be situated, immediately above ideality, in the blank space that appears in the busts and plates of the head. Medical inquirers will find, in this section, an exposition of several natural manifestations which persons untaught by phrenology would confidently attribute to disease. In analysing the sentiment of veneration, Mr. Combe pronounces the following doctrine, which we regard as being very remarkable both for its sublimity and its truth. "As Nature," he says, p. 202, "has implanted this sentiment in the mind and its corresponding organ in the brain, it is a groundless terror to apprehend that religion can ever be extinguished, or even endangered, by the arguments or ridicule of the profane. Forms of worship may change, and particular religious tenets may now be fashionable, and subsequently fall into decay; but, while the human heart continues to beat, awe and veneration for the Divine Being will ever animate the soul; and the worshipper will cease to kneel, and the hymn of adoration will cease to rise, only when the race of man becomes extinct."—Such observations convey a dignified admonition to those silly ones, who persevere in claiming importance to their unreasonable gossipings about the *consequences* of the phrenological doctrines,—as if any but *good* consequences can ever result from *Truth*.

ORDER II. INTELLECTUAL FACULTIES.—These communicate to man and animals, knowledge of their own internal sensations, and also of the external world: their object is to know existence, and to perceive qualities and relations. This order consists of four genera including the five senses,—the knowing,—the judging,—and the reflecting powers: they all perceive objects primarily, and then *know*, *judge*, or *reason on*, their essential or relative attributes.

GENUS I. EXTERNAL SENSES.—After reviewing the notions entertained by philosophers in regard to the functions of the senses, Mr. Combe finds himself authorised, by irrefragable reasons, to conclude that they have been whimsical, extravagant and contradictory. According to his own views, each sense performs its functions in consequence of its own innate constitution alone; and the relations of every sense to external impressions are determinate and subjected to positive laws.

If an odour, for instance, make an impression upon the olfactory nerve, the impression is immediately found to be agreeable or disagreeable; and this feeling arises from the constitution of the sense and the relation established betwixt it and the odorous particles which excite it to activity. The functions of every sense depend only on its peculiar organization; and, hence, no exercise or habit is necessary in order to acquire the special power of any sense: if the organization be perfect, the functions are perfect also; and, if the organization be diseased, the functions are deranged, notwithstanding all preceding exercise. If the optic organs be perfect in newly hatched birds, their sight is perfect, as in chickens, ducks, partridges and quails: if, on the contrary, the organization of the eyes or ears be imperfect, the power of the animal to see or hear is proportionally deficient. In adult persons, vision is deranged, if the eyes be diseased: in the old, the functions of the five senses lose their energy, because the vital power of the organs is diminished.—Nature, Mr. Combe goes on to say, never produced any sense which could not perform its functions, without being supported by another and a different sense,—that, for example, we should not be able to see without feeling, or to hear without seeing. Hence, the propositions appear self-evident, that no sense acquires its functions by means of any other sense, and that any *one* sense cannot be the instrument of producing the sensations experienced by means of *all* the senses collectively. But we must observe, he adds, that different senses may enable us to perceive the same object; and that one sense is more fitted than another to make us acquainted with different objects and their qualities: thus, we may obtain a conception of the figure of a bust, by means of the sense of touch, and also by means of the sense of sight. Each sense, therefore, he concludes, has its peculiar and independent functions, and each is subject to positive laws: but every sense also perceives impressions of which another is not susceptible; and it is in consequence of this circumstance that the external senses *rectify* one another, as metaphysicians phrase it; or rather produce, in our author's words, by their co-operation, an extent of accurate conception which, in an unconnected state, they would have been incapable of producing.

Mr. Combe very justly remarks, that it is a task of considerable difficulty to point out accurately the precise limits of the functions of the senses, because in every act of perception their instrumentality is combined with that of the internal faculties of the mind; and it is not easy to discriminate to what extent the act depends upon the one, and to what extent upon the other. In elucidation of this point, he submits the following considerations. The senses themselves do not *form ideas*: thus, when an impression is made on the hand, it is not the organs of touch which form the conception of the object making the impression; but the nerves in the hand receive the impression, and *a faculty of the mind* perceives the object. Without the nerves of feeling, the internal faculty could not experience the perception; because the medium of communication between it and the object would be wanting; but neither could the hand experience the perception without the instrumentality of the internal faculty, because the nerves of *feeling* do not perform the function of perception. Previously to every perception, therefore, there must be an antecedent impression on the organs of sense; and the whole functions of these organs consist in receiving and transmitting this impression to the internal faculties. The nature of the impression depends on the constitution of the senses, and on the established relation betwixt them and external objects; and, as it is absolutely impossible for the human will to change either the constitution of the senses, or the relation betwixt them and the external world, it is clearly absurd to speak of *acquired* impressions. As the senses, however, are constituted with a determinate relation to external objects, so the internal faculties are constituted with a determinate relation to the organs of sense. In virtue of the first relation, a certain object makes a certain *impression;* and, in virtue of the second, a certain impression gives rise to a certain *perception;* and both depend on *Nature*, and not on the will, nor on exercise and habit. But we must distinguish betwixt the perceptions we experience of external objects, and the inferences concerning their qualities, which we draw by reasoning from these perceptions. All those ideas, which are pure perceptions, are formed *intuitively, on the presentation of objects fitted to excite them.* Inferences from these, on the other hand, are the result of our *reasoning* powers. What are sometimes called "*acquired* perceptions," are merely *habits of reasoning* from the impressions naturally made on the senses; and these *habits* are just as much a part of our *nature* as the original perceptions. The visible and tangible appearances of bodies are simple perceptions, because, after the amplest experience of some of these being deceitful, we cannot in the slightest degree alter our perceptions of them. If, for instance, a picture be painted according to the rules of perspective and the laws of optics, so as to represent a vista in the country, or a long street in a city, we are altogether incapable, when in the proper position for viewing it, of perceiving the surface to be plain: the picture appears to us to represent objects at different distances, and the most determined resolution to *see* them all equally near, is of no avail, although we know that, in point of fact, they really are so. Colour, form, magnitude, and distance, appear to me, says Mr. C. to be objects of *intuitive perception;* and, accordingly, no experience, and no repetition of acts of volition, can alter such appearances, if the refraction of light, state of the eye, and the internal faculties, remain unchanged.

Mr. C. regards the following as a correct mode of ascertaining the limits of the functions of the senses. Whatever perceptions or impressions, received from external objects, *can be renewed* by an act of recollection, cannot depend exclusively upon the senses; because the organs of sense are not subject to the will, and never produce the impressions which depend upon their constitution, except when excited by an external cause. On the other hand, whatever impressions we are *unable to recall*, must, for the same reason, depend on the senses alone. He then illustrates these principles by most appropriate examples: we give one taken from the sense of touch. If, says he, we embrace a square body with the hands, certain impressions are made on the nerves of touch, called sensations, in consequence of which the mind forms an idea of the figure of the body: now, we can recall the *conception* of the figure of the body, but not the sensation which excited it: the conception, therefore, depends on an *internal faculty*, the sensation on the *nerves of touch.* The whole functions of the nerves of touch are to produce the sensation; but the power of conceiving is not in invariable proportion to the power of feeling, but in proportion to the perfection of the internal faculty and the external senses jointly. The perception, however, depends as entirely on nature as the sensation; and the *power* of perceiving the form of the body is not acquired by *experience.* These views of the functions of the senses, which it will be at once observed, correspond perfectly with Dr. Reid's philosophy, are farther illustrated and confirmed by the phenomena which take place, when the organs of sense are diseased. Thus, when the ear becomes inflamed, spontaneous sensations of sound are often experienced: when too much blood flows into the eye, impressions like those of light are felt: when the nerves of taste are diseased, disagreeable savours become perceived: when the nerves of touch suffer excitement from internal causes, a tickling or unpleasant sensation is the result: and, when the muscular system is relaxed by nervous diseases, and flying spasms occur over the body, impressions occasionally arise from these spasmodic affections, so precisely resembling those of touch, that the individual is at a loss to distinguish them.

Our author next proceeds to state briefly the various theories, none of which he holds to be satisfactory, which have been propounded to account for the fact,—*that, though the organs of each sense are double, yet the consciousness of all impressions experienced by the mind is single.* He concludes this statement by remarking, that the mind has no consciousness either of the existence of the organs of sense, or of the functions performed by them. When the table is struck, he says, and we attend to the subject of our own consciousness, we perceive the impression of a sound; but, by this attention, we do not discover that the impression has been experienced by the instrumentality of any organ whatever: hence, the perceptions of the mind are always directed to the objects which make the impressions, and not to the instruments by means of which the impressions are experienced. The instruments perform their function under Nature's care, and are not subject to the will: we should have been distracted, not benefited, by a consciouness of their internal action, when they perform their functions: it is when they are diseased, that we become conscious of their action, and then the consciousness is painful.—But we must, reluctantly indeed, be satisfied with these general remarks; and, in referring our readers to the "System" itself for an acquaintance with these concise and luminous observations of Mr. Combe's on each of the external senses, we would especially impress their importance on the attention of all judicious physiological inquirers.

GENUS II. KNOWING FACULTIES.—These take cognizance of the existence and physical qualities of external objects: they also form ideas and correspond, in some degree, to the "perceptive powers" of the metaphysicians. Their action is attended with a sensation of pleasure, but this, in general, is weak when compared with the emotions produced by the propensities and sentiments: it is a law of the mental economy, indeed, that the *higher* the functions are, the *less vivid* is the emotion attending their active state. This genus comprehends five of the intellectual faculties, and these are named, *individuality*, *form*, *size*, *weight*, and *colouring*. We cannot undertake more at present than to say simply that our readers will find a delightful and instructive exercise, in examining Mr. C.'s philosophy of these faculties and the manifestations of them through their cerebral organs.

GENUS III. JUDGING FACULTIES.—This kind of the intellectual faculties comprises those which discern or distinguish the relations of external objects, and are called *locality*, *order*, *time*, *number*, *tune* and *language*.—Many important discussions are connected with this branch of the subject: we may notice a case, at p. 333, of the *power of using words* having been impaired by disease, while the ability to articulate, and the powers of perception and judgment, remained entire. It is detailed by Mr. Hood, a very intelligent surgeon in Kilmarnock, who enumerates the circumstances from personal observation. The patient, a sober and regular man, aged sixty-five years, and possessed of the ordinary knowledge of written and spoken language, suddenly began to speak incoherently and became quite unintelligible to all those about him; and it was *discovered that he had forgotten the name of every object in nature.* His recollection of *things* seemed to be unimpaired, but the *names* by which *men* and *things* are known, were entirely obliterated from his mind, or, rather, he had lost the faculty by which they are called up at the control of the will. Whether considered as a contribution to the physiology of mind or to pathological science, we do regard this case as being in all respects worthy of serious consideration: the Phrenological Transactions, vol. I. p. 235, and the Phrenological Journal, vol. III. p. 26, contain the history of symptoms, the appearances found on dissection of the brain, and the author's valuable observations on the disease's progress and effects. This paper of Mr. Hood's, indeed, is an excellent illustration of "medical logic:" it, first of all, exhibits a perspicuous detail of facts, and then what the writer regards as the legitimate inductions: it were better for physiology, if equal pains had been expended in determining the *reality* of premises on which important views of man's complicated nature are sometimes inconsiderately fabricated. We entertain a higher opinion, however, of our readers' judgment than to conclude that they will adopt this sentiment on our declaration merely: they will go to the original, and examine this singular question with the patience and candour by which the true desire of knowledge shall ever be distinguished. At the same time, they will find Mr. Combe's general analysis of the functions of Individuality and the other *knowing* faculties, which intervenes between this and the next genus, to be characterized by his usual sagacity and great reach of mind.

GENUS IV. REFLECTING FACULTIES.—These produce ideas of relation; and, in ministering to the direction or gratification of all the other powers, constitute reason and reflection. They are, *comparison, causality, wit,* and *imitation.* In the discussions which unfold the elementary constitution of these faculties and their operations, we are necessarily invited to enter the depths of metaphysical inquiry; but into these, of course, it would not be suitable for us to penetrate. As, however, it is in some degree fashionable within the precincts of certain gregarious coteries, to stigmatize phrenology, as leading to the worst results, viz. "*materialism, fatalism, and the most abominable atheism,*" we would just take occasion to advise a calm perusal of Mr. Combe's concluding paragraphs on Causality, where will be found a true picture of the manner in which phrenologists reason on the nature of Deity and divine things; and, at the same time, the best *philosophical* arguments by which the most "abominable atheism" can be refuted! His article is concluded in these emphatic words. "This faculty *(causality),* therefore, is silent as to the cause of the Creator of man, and cannot tell whether he is self-existent, or called into being by some higher power; but thus far it can go, and it draws its conclusions unhesitatingly, that HE *must exist,* and *must possess the attributes* which it perceives manifested in his works; and, these points being certain, it declares that HE is God *to us;* that HE is *our* creator and preserver; that all his qualities, so far as it can discover, merit our profoundest reverence and admiration; and that, therefore, HE is to *man* the highest and most legitimate object of veneration and worship.

III. MODES OF ACTIVITY OF THE FACULTIES.—All the faculties, when active in a due degree, produce actions good, proper or necessary; excess only of activity occasions abuses. The mere smallness of its organ is not the cause of a particular faculty's giving rise to bad practices; but it may lead to indifference, apathy, or the omission of duty, by leaving another faculty without proper restraint. When in action, from whatever cause, every faculty determines the kind of feeling, or forms the kind of ideas, that result from its natural constitution.

*The propensities and sentiments* cannot be excited to activity by a mere act of the will: thus, by merely willing to experience them, we cannot conjure up the emotions of fear, compassion, veneration: they may enter into action, however, from an internal excitement of the organs, and then the desire or emotion which each produces is experienced whether we will to experience it or not. There are times, as every one must have felt, when involuntary emotions arise within us, for which we cannot account; and such feelings depend on the internal activity of the organs of such sentiments. These faculties, moreover, may be called into activity independently of the will, by the presentment of the external objects naturally destined to excite them; thus, impending danger excites cautiousness and gives an instantaneous emotion of fear; and the contemplation of stupendous objects in nature prompts ideality, and inspires the mind with a feeling of sublimity. In all similar cases, the power of acting, or of not acting, is completely dependent on the will; but the power of feeling, or of not feeling, is not so,—it is perfectly instinctive. This doctrine affords a ready explanation of the unaccountable pleasure, as Hume called it, which the spectators of a good tragedy receive from sorrow, terror, anxiety, and other passions that are in themselves disagreeable and uneasy: it also unfolds the reasons why they are pleased in proportion as they are afflicted, and never feel so happy as when they employ tears, sobs and cries, to give vent to their sorrow, and relieve their breasts, swollen with the tenderest sympathy and compassion. Each propensity and sentiment, then, may be called into activity by presentment of its object; and, when active, the corresponding feeling or emotion attends it, in virtue of its constitution.

Happiness consists in the harmonious gratification of all the faculties; and the very essence of gratification is activity. Thus, the muscular system is gratified by motion, the eye by looking at external objects, and hence pleasure arises. So it is with the propensities; *combativeness* is delighted by overcoming opposition, *destructiveness* by the sight of extinction and the infliction of pain: and with the sentiments; *benevolence* rejoices in the relief of suffering, *hope* in looking forward to a happy futurity, and *cautiousness* in a certain degree of doubtfulness and anxiety. As, therefore, the *degree* of enjoyment corresponds to the *number* of faculties *simultaneously* active and gratified, it follows, that a tragic scene which affords direct excitement to several of the faculties at the same moment, *must be agreeable*, whatever these faculties may be; provided, 1st. It does not, at the same time, outrage any of the other feelings, and 2d. That it does not excite any faculty so intensely as to give rise to pain; just as too much light hurts the eyes, and too much exertion fatigues the muscles. We are to remember, however, that all such internal emotions take place simply in consequence of the constitution of the faculties, and the relation established by nature betwixt them and their objects, without the *understanding* requiring to be imposed upon, or to form any theory, whether the scenes be real or fictitious. If the propensities and sentiments become excessively active from these representations, they may overpower the intellect, and then a temporary belief of their reality will follow and the feelings be the stronger; but, in this case, the strong emotion does not arise *from a previous illusion of the understanding*, but the misconception is the *consequence*, and not the cause of the feelings having become overwhelming. Our readers will find advantage in rightly comprehending this law of our constitution, as it accounts very satisfactorily for several of the phenomena of insanity: thus, for instance, if the organs of combativeness and destructiveness be violently and involuntarily active through disease, madness or fury will ensue: if those of cautiousness become morbidly and permanently active, fear will constantly be felt, and this constitutes melancholy: if veneration and hope be excited in a similar way, the result will be involuntary emotions of devotion, and the liveliest joy and anticipations of bliss, leading when fixed and immoveable to religious insanity. Not unfrequently, a person is insane on a single feeling alone, as hope or veneration; and then, if the false impression which this altered feeling produces, is admitted as correct, the deductions of the mind from it, as well as the general conduct of the patient, are rational and consistent: thus, says our intelligent author, p. 374, a person insane in self-esteem sometimes imagines himself a king: grant this to be the case, and he speaks and acts as becomes a king, and shews considerable tact and consecutiveness of judgment; this proceeds entirely from the organs of intellect remaining sound, and that of self-esteem having become diseased. In such cases, the most eloquent arguments addressed to the understanding, as a means of removing the disease, must necessarily prove unavailing; for the malady consists in an *unhealthy action of the organ* of a sentiment, and so long as the disease lasts the insane feeling will remain: it will be cured by a speech when the gout yields to a good story.

Nevertheless, the propensities and sentiments may be excited to activity, or repressed, *indirectly*, by an effort of the will: thus, the knowing and reflecting faculties form ideas; now, if these be employed to conceive internally the objects fitted by nature to excite the propensities and sentiments, the latter will acquire activity in the same manner, but not in so powerful a degree, as if their appropriate objects were externally present, and the vivacity of the feeling will be in proportion to the strength of the conception and the energy of these mental powers. If, for example, we conceive inwardly an object in distress, and benevolence be powerful, compassion will be felt and tears will sometimes flow from the emotion produced: if, in like manner, we wish to repress the activity of ideality, we cannot do so by willing that the sentiment be quiet; but, by conceiving objects naturally adapted to raise other sentiments, as veneration, fear, pride, or benevolence, these faculties will then be excited and ideality will sink into inactivity. Hence it is, that he who has any propensity or sentiment predominantly active from internal excitement of its organ, will have his intellect filled with conceptions fitted to gratify it: in other words, the habitual subjects of thought in the mind are determined by the faculties which are predominantly active from *internal* excitement. These principles, from their explaining at once the great variety of tastes and dispositions among mankind, should be particularly attended to in the applications of medical as well as moral philosophy; for, in no two individuals, is exactly the same combination of organs to be found, and hence every one is inspired with feelings in some degree peculiar to himself, and desires objects fitted for their especial gratification. As the propensities and sentiments, in fine, do not form ideas, and as it is impossible to excite or recall the feelings or emotions produced by them, *directly* by an act of the will, it follows, says Mr. C. that these faculties have not the *attributes* of perception, conception, memory, and imagination. They have the attribute of sensation alone, *i. e.* when they are active, a sensation or emotion is experienced: hence, sensation is an accompaniment of the activity of all the faculties, and of the nervous system in general; but sensation is no faculty in itself,—it is purely an attribute or characteristic disposition.

The *knowing* and *reflecting* are subject to laws different from those of the *propensitive* and *sentimental* faculties: they form ideas and perceive relations; are obedient to the will or rather constitute will themselves; and they minister to the gratification of the other faculties which only feel. They may be active, also, from internal excitement, and then the kinds of ideas which they are fitted to form are presented involuntarily to the mind: thus, the man in whom causality is powerful and active, reasons while he thinks without an effort; and he, in whom wit is energetic, feels witty conceptions flowing into his mind spontaneously, and even at times and places when he would wish them not to appear. Another modification of excitement may be communicated to these faculties by the presentation of the external objects fitted to call them into activity; and another is desirable from an act of volition. At this stage of his labours, Mr. Combe is led to consider the important doctrines of perception, conception, spectral illusions, dreaming, imagination, memory, judgment, consciousness, attention, association, passion, pleasure and pain, joy and grief, sympathy, habit, and taste. We feel vexation in being obliged to confine ourselves to Mr. Combe's definitions; but we can assure our readers that they will find his discussions in illustration of these mental states, to be as instructive as they are excellent.

1*st. Perception.* This is a simple act of the mind by which, on the presentation of external objects, these objects are known *to have existence:* it constitutes the *lowest degree of activity* of the knowing and reflecting faculties; and, if no idea is formed when the object is presented, the observer is destitute of the power of manifesting the faculty whose function is to perceive objects of that kind: it constitutes a *mode of action* of these faculties, but is not a separate faculty itself.

2*nd. Conception.* When the knowing and reflecting organs are powerfully active from internal excitement, whether by the will or by natural activity, then ideas are vividly and rapidly formed, and the act of forming them is styled *conception:* if this act be executed with a very high degree of vivacity, it is called *imagination;* but each faculty performs the act of conception in its own sphere. When, moreover, any of these organs is internally active, the mind conceives, or is presented with ideas of, the objects which it is fitted to perceive: but, if this internal activity become morbid, through disease of the organs, these ideas become fixed and remain involuntarily in the mind, and thus give rise to insanity.

3*d. Spectral Illusions.* If several of these last organs become active through internal excitement, they produce involuntary conceptions of outward objects invested in all the attributes which usually distinguish reality. The organs of the knowing faculties, and that of wonder, seem to Mr. C. to be the chief seats of these diseased perceptions; and this, he thinks, appears obvious from the descriptions of the illusions themselves: in demonstration of this view, he adduces a very remarkable history from the Phrenological Journal which, as well as the concluding part of this section, we entreat our metaphysical physicians to peruse with candour, and to examine with that degree of attention a question so momentous evidently demands.

4*th. Dreaming.* If, says our author, the greater number of the organs remain inactive, buried in sleep, and two or three, from some internal excitement confined to themselves, become active, these excited organs will present the mind with corresponding conceptions: and, being separated in their action from the other organs, which, in the waking state, generally co-operate with them, the result will be the creation of disjointed and fantastic impressions of objects, circumstances, and events; in short, he adds, all the various phenomena of dreaming. Hence, every thing that disturbs the organization of the body may become the cause of dreams; a heavy supper, by encumbering the digestive powers, affects the brain by sympathy and thus originates the spectres dire which then assail the sleeping fancy: fever, too, by keeping up a morbid excitement in the whole system, sustains the brain in a state of uninterrupted activity, and thereby prolongs the sleeplessness which attends the higher, and the disturbed dreams that accompany the lower, degrees of that disease. In the same way, another familiar fact relative to the mind may be explained,—if, when awake, we have been excessively engaged in any particular train of study, it haunts us in our dreams. During the day, the organs of the faculties chiefly employed, were maintained in a state of action, intense and sustained in proportion to the mental application: by a general law of the constitution, however, excessive action does not subside suddenly, but abates by insensible degrees: hence, on going to sleep, so much activity continues to stimulate the organ, that the train of ideas goes on till, after long action, it at last entirely subsides. Mr. Combe finds that dreams in different individuals have most frequently relation to the faculties whose organs are largest in the brain; and he presumes, but does not state it as a fact, that persons in whom cautiousness is small and hope and benevolence large, will, when in health, enjoy brilliant and happy dreams; while others, in whom cautiousness is very large and hope small, will be wading in difficulties and woe: this new doctrine will not be lost, we presume, on the enlightened medical inquirer. Some years ago, Mr. A. Carmichael of Dublin suggested the idea that sleep may be the occasion when the waste of substance in the brain is repaired by the deposition of new particles of matter. There is no direct evidence of the truth of this conjecture; but the brain, like every other part of the animal structure, is furnished with sanguineous, and secerning, and absorbing vessels; and, like them also, is exposed to the influences of excessive deposition or absorption. That its waste should be repaired, therefore, is a fact of necessary inference; and, that the period of sleep, when the mental functions are suspended, would be particularly suitable to this operation, is also matter of very plausible conjecture; but, here the matter must rest, till farther observation and facts enable us to advance towards a more certain philosophy.

5th. *Imagination.* Conception is the cool and methodical representation of things absent to one's self or to others: imagination is the *impassioned representation* of the same things, and not merely in the forms and arrangements of nature, but in new combinations formed by the mind itself: thus, perception may be viewed as the *first* or lowest, conception as the *second*, and imagination as the *third* degree of activity of the knowing and reflecting faculties. In other words, it is just intense, glowing, forcible conceptions proceeding from a great activity of the intellectual powers, and these conceptions not confined to real circumstances, but embracing as many new combinations as the faculties are capable of commanding.—Mr. C. concludes this sketch with an ingenious disquisition on the meaning of the indefinable word "Fancy," to which we must only advert, and proceed.

6th. *Memory.* As the mind has no power of calling up, into fresh existence, the emotions experienced by means of the propensities and sentiments, by merely willing them to be felt phrenologists therefore, hold the doctrine, that *memory is not possessed by these faculties.* The ideas formed by the knowing and reflecting faculties, however, can be recalled by an act of recollection, *and they are, therefore, said to have memory:* memory, consequently, is nothing else than a mode of activity of the knowing and reflecting organs. In conception and imagination, which also result from internal activity, *entirely new* combinations of ideas are formed; and the ideas themselves are recalled, not only without regard to the time or order in which they had previously existed, but even without any direct reference to their having at all existed before. Memory, on the other hand, implies a new conception of impressions previously received, attended with the idea of past time and consciousness of their former existence; and this new conception follows the order of the events as they happened in nature. Each organ, in fine, enables the mind to recall the impressions which it served at first to receive; and, hence, there may be as many *kinds of memory* as there are *knowing and reflecting organs.* The doctrine, moreover, that memory is only a degree of activity of the organs, is illustrated by the phenomena of diseases which particularly excite the brain: hence, occasionally, under the influence of disease, the most lively recollection of things will take place, which had entirely escaped from the memory in a state of health. Mr. Combe exemplifies this by a remarkable pathological history, in the examination of which, we doubt not, our readers will be actuated entirely by the spirit of patient inquiry after truth.

7th. *Judgment.* This is the perception of relation, or of fitness, or of the connexion betwixt means and an end, and belongs solely to the *reflecting* powers: these have perception, imagination, and memory, as well as the *knowing* faculties; but judgment is the decision or inference of the *reflecting* faculties upon the *feelings* induced by the propensities and sentiments, and upon the *ideas* furnished by the knowing faculties. To judge of the proper line of conduct to be followed in the affairs of life, therefore, it is necessary to *feel* correctly as well as to reason deeply; or rather, it is *more* necessary to feel rightly than to reflect: hence, if an individual possess very powerful reflecting faculties and, at the same time, be deficient in conscientiousness, he is like a fine ship wanting a helm, liable to be carried from her course by every wind and current. Mr. C. draws an apt illustration of this doctrine from the character and actions of Lord Bacon, and shews, by example, the results of neglecting its principles in the affairs of ordinary life, and especially in that important regulation which requires the *unanimity* of juries in the trial of civil causes: a proposition, he concludes, to which nine men out of twelve would *voluntarily* assent, would be nearer truth than one modified by mutual concessions to conciliate, *but not to satisfy*, the whole.

8*th*. *Consciousness*. This means the knowledge which the mind has of its own operations, but gives no intimation of the existence of its organs: it reveals to us only the operations of our own minds, leaving us entirely in the dark regarding the mental affections of others, where they differ from our own. Hence, by reflecting on consciousness as the means of studying the mind, we can discover nothing concerning the organs by which the faculties act, and run great danger of forming erroneous views of human nature, by supposing mankind *in general constituted exactly like ourselves*.—Under this head, Mr. C. introduces a case of what is called a *divided consciousness or double personality*—exhibiting in some measure two separate and independent trains of thought and two independent mental capabilities, in the same person: it involves several most curious philosophical questions, which Mr. Combe, with his usual candour, professes his inability phrenologically to explain: it is an account of *diseased* manifestations which, of course, are contrary to nature, and, by consequence, do not come under the cognizance of phrenology, as this professes to be nothing more than the faithful interpreter of Nature's laws.

9*th*. *Attention*. This is not a *faculty* of the mind, but consists merely in the application of the knowing or reflecting faculties to their objects: it is, indeed, a mere *act* of the different intellectual powers, and not the *attribute* of any *particular* power, established exclusively for its production.

10*th*. *Association*. Mr. Combe affirms that the metaphysicians have erred, and must ever err, in endeavouring, by reflecting on their own consciousness, to discover universal laws, by which the succession of ideas in mankind in general will be regulated. They imagine our thoughts to follow each other, in an established order of succession; and, in their peculiar way, have attempted to find out the circumstances which determine the *order* and the *causes*, in virtue of which one idea introduces another into the mind. Our author, however, demonstrates the fallacy of this position, by shewing, as an illustration, the impossibility of ascertaining the laws that regulate the succession of notes emitted by an Æolian harp, and of controlling the ever-varying actions of the inconstant air, which form the causes producing the notes. Ideas, he says, are affections of the human faculties, just as notes are affections of the strings of the harp; and they arise from impressions on the various powers of the mind; but there is as little regularity in the order in which they are received, as in the breathing of the air on the strings of the harp: if harps, then, *may* vary in structure, human beings *do* positively differ in the relative strength of their faculties, so that one possesses several strong, which in another are weak; or, in one, all the powers are active, and in another sluggish. Hence, the same impression must produce very different effects, or introduce very different ideas into minds dissimilarly constituted: how then, Mr. C. asks, amid such a countless variety of causes, can similarity of effects be expected? His illustrations of this abstruse doctrine are beautifully appropriate; but we cannot do more than stay to solicit an impartial examination of his philosophy. In conclusion, he says, in studying the laws of association we must go beyond the ideas themselves, and consider the faculties which form them: thus, we shall find the individual who has the *reflecting* faculties most powerful, associating ideas according to the relation of necessary consequence; we shall find him who has the *knowing* faculties most powerful, associating ideas according to the relations of time, place and circumstances; and, in every case, we shall find each individual associating those ideas with most facility, and recollecting those ideas most perfectly, which minister to the gratification of his most powerful propensities and sentiments. He gives this illustrative example: if we place a number of persons on a hill-top, Arthur's seat, for instance, overlooking a picturesque country and the sea, and bid each declare his thoughts;—we shall find that one, with ideality predominant, will think of the magnificence of Nature, the boundless extent of the ocean, the vastness of the mountains; and, on recalling the scene, these ideas and emotions will be associated with it in his mind:—another, with great causality and constructiveness, and little ideality, will admire the skill which he sees displayed in laying out the fields, and in constructing the houses and the ships:—one, with benevolence large, will think of the happiness enjoyed by the people who inhabit the plain:—another, with acquisitiveness active, will think how the various branches of industry will pay:—and one, with a strong veneration, will probably take occasion to admire the greatness and goodness of God. Now, each of these individuals, on the mention of the hill, shall afterwards experience a train of ideas corresponding to the first impressions which he received on the top, and nothing can be more dissimilar than these: as well, therefore, may we expect, by studying the forms and lines of the clouds that flit along the sky to-day, to be able to discover laws by which their succession shall be regulated to-morrow; as, by reflecting on the ideas that pass in one mind, to discover links of association by which ideas in the minds of mankind in general will be uniformly connected and introduced in a determinate succession.

11*th*. *Passion* is the highest *degree of activity* of every faculty; and the passions are as different as the faculties. There can be no such thing as *factitious* passions: man cannot alter his nature; and every object he can desire must be desired in consequence of its tending to gratify some natural faculty: this doctrine should be exceedingly available in the applications of medicine.

12*th*. *Pleasure* and *Pain* are affections of every faculty: they are the result, not the cause, of the particular faculties. Every faculty, when indulged in its natural action, feels pleasure; when disagreeably affected, feels pain: thus, one person delights in pardoning offences, another in taking revenge.

13*th*. *Joy* and *Grief*. Each propensity desires to attain its object, and this attainment affords to the mind a feeling of gratification: thus acquisitiveness desires wealth, and the *obtaining of wealth* gratifies the propensity: this is attended with pleasing emotions, and these emotions constitute *joy*: again, the *losing of wealth* deprives the propensity of its natural object; this, therefore, is accompanied with painful sensations, and these make up *grief*.

14*th*. *Sympathy* is defined to be a fellow-feeling in one person, with emotions experienced by another; and, in a very philosophical sketch contributed to the author by his brother Dr. A. Combe, the laws which regulate the activity of the mental faculties in sustaining this affection, and the circumstances most favourable to its occurrence, are explained with great conciseness and perspicuity. The doctrine of sympathy leads to many important practical results: the generous mind will retrace Dr. C.'s exposition of it with advantage and delight.

15*th*. *Habit*. Every voluntary action is a manifestation of some one or more faculties of the mind: now, before this action can be done, the faculty on which it depends must be possessed; and, the stronger the faculty, the greater will be the facility with which the individual will do the thing at first, and with which he will learn to repeat the doing of it. Habit is the power of performing such voluntary action, readily and easily; and this power is acquired or improved by frequent doing the voluntary action: thus, before the habit of practising particular branches of art and science can be acquired, the organs on which they depend must be largely possessed; and, being so, the habit results spontaneously from exercising the powers. Exercise enable the organs to act with greater facility; and, it is in this way, that the *real* effects of habit on the mind, which are important, can be explained; but still the organ must possess a certain, and even a considerable degree of natural power and activity, to render it susceptible of the exercise by which habit is formed.

16*th*. *Taste*. Every act of the mind must be a manifestation of some faculty or other; and every act must be characterised either by good or bad taste, or be wholly indifferent in this respect: an inquiry into the origin of bad taste, will lead us to distinguish its opposite, or correct taste. Bad taste, then, appears to arise from an excessive or inappropriate manifestation of any of the faculties: those poets, for instance, are guilty of very bad taste in the passages of their writings where they exhibit the passion of love in all the grossness of an animal feeling. Faults in taste, however, proceed not only from unbecoming manifestations of the lower propensities, but also from an inordinate expression of the sentiments and intellectual faculties: thus, when abused, ideality itself, which is undoubtedly the fountain-head of beauty, may degenerate into bombast, rant, exaggeration, and the wild extravagances of drunken sublimity. Excess of vanity and the tendency to engross conversation, proceeds from over-active love of approbation and self-esteem: the disposition to wrangle, dispute, and contradict, springs from an excessive manifestation of combativeness: and, from an improper exercise of secretiveness and love of approbation, arises the practice of flattering, and uttering a profusion of agreeable things to persons whom we do not esteem, but wish to please. On the other hand, the most exquisite mental manifestations are those which proceed from a favourable combination of the whole faculties, and in which each contributes a share of its own good qualities, and is restrained by the others from running into excess or abuse. Good taste, therefore, in being the result of the harmonious action of the faculties, is susceptible of great improvement by cultivation. An author, says Mr. Combe, will frequently reason as profoundly, or soar as loftily, in his first essay, as after practice in writing for twenty years; but he rarely manifests the same tact at the outset as he attains by subsequent study and the admonitions of a discriminating criticism. This is the case, because reasoning depends on causality and comparison, and lofty flights on ideality; and, if these faculties be eminently possessed, they execute their functions intuitively, and carry the author forward, from the first, on a bold and powerful wing: but, as taste depends on the adjusting, the suppressing and elevating, the ordering and arranging, of our thoughts, feelings and emotions, so as to produce a general harmony in the manifestations, it is only practice, reflection, and comparison with higher standards, that can enable us successfully to approximate to excellence; and even this can be gained only when the organs are by nature equally combined; for, if the balance preponderate greatly in any particular direction, no effort will produce an exquisite taste.

Mr. Combe next gives the doctrine of the *effects of size in the organs on the manifestations of the faculties.*—Size in the organs, it has been stated already, is, *cæteris paribus*, the measure of power in the faculties: as size, therefore, is an indispensable requisite to power in the mind, no instance ought to occur of an individual who, with a small brain, has manifested clearly and unequivocally great force of character, animal, moral and intellectual:—*general* size, however, cannot be the indication of *particular* power; we must always look for the *power* in the *direction* of the size: the union of *size* and *activity* in an individual, as Homer and Shakespeare, constitutes the perfection of genius.

Next in order is the section on *combinations in size, or the effects of the organs, when combined in different relative proportions*, with rules for estimating the effects of differences in relative size, occurring in the organs of the same brain; and appropriate practical illustrations which, however, our limits oblige us to pass. These are the rules.

*Rule 1st.* Every faculty desires gratification with a *degree of energy* proportionate to the *size* of its organ; and those faculties will be habitually indulged, the organs of which are largest in the individual.

*Rule 2d.* As there are three kinds of faculties, animal, moral and intellectual, which are not homogeneous in their nature, it may happen that several large animal organs are combined in the same individual, with several moral and intellectual organs highly developed: the rule then will be, that the lower propensities will take their *direction* from the higher powers; and such a course of action will be habitually followed as will be calculated to gratify *the whole faculties whose organs are large.*—The examples given in illustration of this rule are truly and profoundly philosophical.

*Rule 3d.* Where all the organs appear in nearly equal proportions, the individual, if left to himself, will exhibit opposite bearings of character, according as the animal propensities or moral sentiments predominate for the time: he will pass his time in alternate sinning and repenting. If external influence is brought to operate upon him, his conduct will be greatly modified by it: if placed, for instance, under severe discipline and moral restraint, these will cast the balance, for the time, in favour of the higher sentiments; if exposed to the solicitations of profligate associates, the animal propensities will probably obtain triumphant sway.

*Combinations in Activity.* Where several organs are large in the same person, they have a natural tendency to combine in activity and to prompt him to a line of conduct calculated to gratify them all: where, however, all or the greater part of the organs are possessed in nearly equal proportions, important practical effects may be produced, by establishing combinations in activity among particular organs or groups of organs. It is in virtue of this principle that education produces its most important results, occasioning the practical conduct of individuals to be very different, in consequence of the difference of their training. In viewing the heads of the higher and lower classes of society, we do not perceive the animal organs preponderating in point of size in the latter, and those of the moral sentiments in the former, in any very palpable degree: the high polish, therefore, which characterises the upper ranks, is the result of sustained harmony in the action of the different faculties, and especially in those of the moral sentiments, induced by long cultivation: the rudeness observed in some of the lower orders proceeds from a predominating combination in activity among the lower propensities; and the awkwardness that frequently characterises them, arises from the propensities, sentiments and intellect not being habituated to act together. If, however, an individual is very deficient in the higher organs, he will remain vulgar, in consequence of this defect, although born and educated in the best society, and in spite of every effort to communicate refinement by training; while, on the other hand, if a very favourable development of the organs of the higher sentiments and intellect is possessed, the person, in whatever rank he moves, will have the stamp of Nature's nobility.—The student who attends to the doctrine of the primitive faculties and their combinations, will perceive the reasons *why* phrenology does not enable us to predict *actions*.

Our author's next section is long, and treats of a subject possessing the highest degree of importance: it is titled, "*On the coincidence between the natural talents and dispositions of nations, and the development of their brains.*" We cannot do it justice in the limited sketch to which we must confine ourselves in this article; and shall therefore, state merely one of Mr. Combe's incontrovertible conclusions, viz. that national character, instead of being modified by climate, religious and political institutions, and other external circumstances, is absolutely dependent on particular innate dispositions and talents concomitant invariably with a particular conformation of brain. Those, he says, who contend that institutions come first, and that character follows as their effect, are bound to assign a cause for the institutions themselves: if these do not spring from the native mind, and are not forced on the people by conquest, it is difficult to see whence they can originate.

Equally important and beautiful are the doctrines inculcated by Mr. Combe in his chapter intitled, "*On the harmony of the mental faculties with each other, and with the laws of physical nature.*" In this, he shews to a demonstration, that the dictates of each of the faculties proper to man are in harmony with the dictates of all the others; and that the Creator has established such relations between the human mind and the external world, that happiness, benefit, or advantage, is the natural result of actions approved by the moral sentiments and intellect; and evil or suffering the natural consequence of all manifestations of the lower propensities in opposition to the dictates of the higher powers. So proper, indeed, and so determinate is this harmony, that it is possible, in general, to gratify *the whole faculties,* without abusing any, and that the result of such gratification will be satisfaction and real enjoyment, highly favourable at the same time to morality and religion. To be able to find out the line of conduct calculated to lead to this result, each individual must know, 1st. The nature of his own faculties, their legitimate uses, and their abuses: 2d. The relations of these faculties among themselves; and 3d. Their relations to external beings and objects. Its extreme elegance of diction, but more particularly the profound penetration and practical wisdom displayed in its philosophy, will necessarily obtain for this section the reader's highest consideration.

*On Insanity and Criminal Legislation.* Future ages, says Mr. C. will be surprised when they learn that, in the 19th century, metaphysical professors in every college lectured upon the mental powers, and were listened to by attentive hearers; but that, when the manifestations of mind became deranged, the knowledge of these persons was conceived to bear so small a relation to the case, that they were passed by, and a physician or surgeon, probably altogether unacquainted with metaphysics, had the disordered mind committed to his administration for a cure. If the philosophy of mind, in its sound state, were truly founded in nature, such a separation of theory from practice could not occur: the fundamental error originates in the neglect, by the metaphysicians, of the influence of the organization with which the mind is connected. They study the mind as if it were a disembodied spirit: but a spiritual essence cannot become diseased and be cured by medicine; the organs alone are subject to malady; and hence, whenever the action of these corporeal instruments becomes deranged and unusual manifestations occur, a new element as it were starts into play, not known or admitted in the metaphysical systems, and their whole rules, principles and theories become inapplicable. The phrenologist, on the other hand, studies the mental powers in connexion with the organization: he ascertains the healthy functions of the faculties and organs; and, when they become diseased, the phenomena appear to him natural and easy to be accounted for.

With some, it has been the practice to treat madness, or fury, by bleeding and general depletion; and cases of melancholy by administering bark, wine, and other tonics and stimulants: now, the phrenologist would be led to suspect that the *cause* of both these diseases may frequently be the same, or that inflammatory action, affecting the *organs* of combativeness and destructiveness, may produce the symptoms denominated fury; and that some cause affecting the *organs* of cautiousness, conscientiousness, and veneration, may give rise to the symptoms designated melancholy. It is obvious, of course, that all remedies ought to have reference to the *cause* of the disease, and not vary merely on account of the symptoms: it would, therefore, be preposterous to treat the affection of the one set of organs by depletion and that of the other by tonics. A correct knowledge of phrenology will be of some service in preventing the physician from mistaking difference of *symptoms*, arising from the same cause, attacking different parts of the brain, for actual difference of *disease*; and it will tend to introduce system and consistency into his administration of remedies. Diseases of the organs of the mind differ from affections of other organs in this, that they are susceptible of great alleviation from moral treatment. The basis of a rational moral treatment must be to avoid every circumstance and idea that can by any possibility irritate and excite the diseased feeling or intellectual faculty: now, the physician, who knows the boundaries of each faculty when in health, its range of objects and interests, and who discriminates correctly the precise powers impaired, must obviously have it in his power to avert all prejudicial influences from the patient, much more than he who possesses either no knowledge, or an imperfect knowledge founded only on vague conjectures, concerning the faculties in health; who does not believe in the existence of separate organs and powers; and who has neither theory nor principle, in regard to the mind, on which he can ground his proceedings. The physician, however, who is acquainted with the true science of mind, and possesses sufficient mental tact to avail himself of its aid, will be able to discover the range of his patient's desires and aversions, and of his intellectual strength and weakness; and, what is of no mean importance, be able to satisfy the patient that he understands his disease.

As we intend returning to an enlarged consideration of this subject, in a review of Dr. Spurzheim's work on insanity, we shall satisfy ourselves, for the present, with upholding the doctrines promulgated in this section of Mr. Combe's to the reader's gravest attention: they are of vital importance to every medical inquirer.—We would just add the following notes and pass on. From a profound knowledge of the question, he states, that a large and active brain, well developed in the moral and intellectual regions, is indispensable in the person himself who undertakes the practical direction of the insane, to enable him to apply the principles of phrenology to their full account; for it is undeniable, he adds, that many men of respectable attainments possess no tact for treating diseased feelings, sentiments, and intellectual powers, even although the constitution of their organs were known to them as matters of science, and besides, they possess no mental resources to enable them to call forth and support in action the faculties of their patients, by the exercise of their own. The resident director of an asylum, he proceeds to say, ought to be a man of genius, largely endowed by nature with the propensities, sentiments, and intellectual powers in his own mind; and, moreover, well instructed in medicine, phrenology, and the collateral branches of knowledge. Such a person would possess all the chords of mind powerful in himself, and could touch all the varieties of them in his patients: he would enjoy that force of character and buoyancy of spirit which have immense influence in calling out latent energies, and even in soothing irritated feelings.—Physicians, in judging of insanity, ought never to lose sight of the important fact, first revealed by phrenology, that the organs of some faculties and, consequently, their manifestations, may be diseased or deficient, while others remain entire: this of itself removes every difficulty.

*Objections to Phrenology.* Under this head, Mr. Combe examines the objections so frequently, and we may add, so senselessly, urged against his favourite science,—*that which accuses it of leading to materialism; and that which rests on the alleged fact of the brain having, in various instances, been wounded or destroyed in whole or in part, without in any degree impeding the usual operations of mind.* The last of these questions is investigated in an elaborate paper furnished to the "System" by Dr. A. Combe, who succeeds completely in removing the objection: his reasonings to us seem unanswerable, and unfold views which the physician can apply to the best purposes: the essay altogether is satisfactory and excellent. As, however, we took a pretty comprehensive view of his arguments, in a former number of our Journal, our reverting here to the subject will not be expected. In regard to the shallow and unphilosophical statement, that phrenology leads to "materialism, fatalism, and the most abominable atheism," it has been so often refuted that we should have supposed nothing but the most childish obstinacy could have advanced it as an anti-phrenological argument, had we not of late discovered that Sir William Hamilton of Edinburgh, who is a superb scholar and a sublime gentleman, has satisfied the Royal Society of the Scotch Metropolis that the objection is founded on truth, and of course susceptible of establishment beyond all contradiction. As Sir William is pledged to publish, for the benefit of misguided philosophers, the exquisite Essay in which this high object is attained, we shall expect with anxiety and desire, the full completion of his philanthropic designs; and, in the mean time, we would suggest, in anticipation of his glory, that the phrenologists, as an atonement for their insolence and stupidity, should prepare themselves to erect a monument on Arthur's Seat, representing a wrestling-match, in which the "doughty" Baronet, as a Titan, overcomes and "crushes" a Pigmy.

In passing, we recognise the aptness of the five articles which constitute the appendix to Mr. Combe's system, and proceed to give a summary view of the more important questions in the science of mind that may be considered as being established by phrenology, with the object of showing more impressively the advantages it seems destined one day to confer on mankind: it demonstrates, then,

1st. That the brain is an aggregate of organs, and, as an organic system, constitutes exclusively the organ of the mind.

2d. That the mind possesses a number of distinct innate faculties, each of which is dependent on a particular material organ in the brain for its manifestation; the *power* of each faculty's manifestation being, *cæteris paribus*, in proportion to the *size* of its cerebral organ.

3d. That these faculties and their corresponding organs are divided into three great classes,—propensities, sentiments, and intellect, between which faculties themselves, and between them and the external world, God has established the most perfect harmony of relation.

4th. That *faculties*, and not *ideas*, are innate, or implanted by Nature.

5th. That attention, perception, memory, and imagination, are not primitive faculties of the mind, but only *modes of activity* of all or any of the intellectual faculties; mere *acts* of the mental powers.

6th. That there is an infinite variety among individuals in their respective endowment of the primitive faculties: hence the differences among men are original and innate.

7th. That these original differences descend, by the laws of propagation, from parents to children; and that it is upon this principle chiefly that national character depends,—feebleness of character being caused by the inheriting from nature a small brain, and energy of character from a large.

8th. That a distinctive character of the sexes consists particularly in the disposition of the propensities of amativeness and philoprogenitiveness, and in general size of brain.

9th. That the essential distinction between man and the lower animals depends on his possession of peculiar organs of the sentiments and of the reflecting faculties.

10th. That man possesses a natural sentiment—*veneration*—leading him intuitively to the worship of a God.

11th. That man has an innate moral sense, depending chiefly, though not solely, on his sentiment of conscientiousness.

12th. That the existence of the faculties of adhesiveness, acquisitiveness, secretiveness, love of approbation, benevolence, conscientiousness and intellect, proves that a state of society or civilization is *natural* to man.

13th. That we may determine, *à priori*, the education most suitable to be given to, and the professions best adapted for, different individuals.

14th. That insanity is, *in every case*, a bodily and not a mental malady; and that the *seat* of the disease is *exclusively in the brain*, or in some particular part of it: *let not prejudice deprive any physician of the immense advantages to be derived from a thorough knowledge of this principle.*

15th. That the cause of partial insanity proceeds from one or more organs becoming diseased, while every other organ, and particularly those of the intellectual faculties remain in a state of perfect sanity.

16th. That the causes of idiotcy, partial or total, arise from the deficiency of size or structure in all, or any of the organs.

17th. That the phenomena of dreaming are dependent on the *activity of some only* of the organs, while profound sleep consists in the *repose of them all.*

18th. That the causes of the independence of nations are dependent chiefly on endowment of the higher sentiments, and not on particular forms of government,—free institutions being the effects, and not the causes of liberty.

Let these suffice, as a specimen of the phrenological principles.—Enough, we trust, has been said in this article to procure for Mr. C. and his philosophy, a fair and courteous hearing. We might have expatiated at great length on the utility of his science, in its applications to the purposes of education, legislation, political economy, criminal jurisprudence, history, legal and theological elocution, and above all to the true philosophy of Medicine; but we have abstained from this indulgence, in the belief that the foretaste of an intellectual luxury we have provided for our readers will stimulate them to desire the enjoyments of a full repast.

We can dedicate but a very small space to the notice of Dr. Spurzheim's work, as it consists almost entirely of prints of distinguished characters, with historical notices of their lives, and some short remarks on their physiognomy, as evinced by the form and development of the brain rather than the face and features.

That the forms of things very frequently indicate the qualities, there can be little doubt—and that even in inanimate objects. There is a physiognomy of the heavens, as evinced by the clouds and skies—the husbandman judges of the soil by its aspect—and the healthy or diseased condition of plants is evinced by external signs. Again, the qualities of animals are often exhibited in their physiognomy. Celerity is visible in the configuration of the roe—sluggishness in the bear—innocence in the lamb—activity in the monkey. The physician speaks of the consumptive or apoplectic conformations of the body—and not only of general but of particular diseases by the countenance. "Finally," says Dr. S. "the affective and intellectual characters of man, in the healthy and diseased states, are proclaimed by physiognomical signs." In looking about us we distinguish, as by intuition, the benevolent, candid, and modest individual, from him who is cruel, artful, and haughty.

Some physiognomists have looked for the signs of men's dispositions over the whole body, while others search for them in particular parts of it only, as in the face and forehead. That it is not in the general figure of a human being we are to look for the dispositions of the mind, is abundantly evident. Æsop and Socrates are proofs that a fine form is not necessary to greatness of talent or generosity of soul. Lavater himself was obliged to admit that ungainly forms are sometimes combined with honesty of character—while individuals, beautiful and well proportioned, are occasionally deceitful. The physiognomical signs therefore of the affective and intellectual faculties, as innate dispositions, are only to be sought for in the size and organic constitution of the cerebral parts.

In illustration of this position, Dr. G. has given us a great many heads, chiefly copied from plates in the *Cabinet d'Estampes* of the Royal Library at Paris, the historical notices of the individual characters being taken from the best authorities. These portraits and characters certainly afford an instructive and entertaining elucidation, and perhaps confirmation of Dr. Spurzheim's views. We can only glance at a very few of them.

*Emperor Caracalla.* This, when examined on phrenological principles, is one of the most ignoble configurations of a head which it is possible to conceive. The basilar region contains a great mass of brain, whilst the sincipital is very small and contracted. The head is low and wide, particularly above and behind the ears. The forehead is narrow, and by no means elevated. In short, the organs of the lowest propensities predominate over those of the moral and religious sentiments, and of the reflective faculties, which are all exceedingly defective. History has but too amply confirmed these phrenological views. Caracalla was fierce, haughty, hypocritical, licentious, selfish, implacable, detestably cruel. He wished to possess all the money of the empire. His understanding was limited, and he continued ignorant, in spite of all the care expended on his education. He lived amid debauchery himself, and punished it in others. He endeavoured to put his father to death—murdered his brother in the arms of his mother—and then prostrated himself before the images of the tutelary deities, averring that he had just escaped the treacherous attempts of him whom he assassinated!

*Zeno, the Stoic.* This portrait, from an antique bust, presents a cerebral organization which must excite the admiration of the phrenologist. The frontal and sincipital regions predominate greatly over those of the basis and occiput. The organs of benevolence, veneration, firmness, conscientiousness, ideality, and of the reflective faculties are eminently large, whilst those of the animal feelings are subordinate. Such a brain is incompatible with grovelling and unworthy conceptions. It procaims superiority in the moral character, and constitutes the sage. Zeno's character and intellectual dispositions exactly agreed with the indications furnished by his bust. He believed in one God, the soul of the world, and had great confidence in the instinct of Nature. His moral principles were severe in placing happiness in virtue, he insisted on the same bearing both in pleasure and pain—and contentment in every situation, both of adversity and prosperity.

These two sketches of the phrenological indications and historical characteristics of individuals, will give the reader some idea of the fund of interesting matter contained in this work of Dr. Spurzheim's.

There can be no doubt that the organs on which the dispositions of a man depend, must lie deeper than in the features of the countenance; and that the surest way of ascertaining these dispositions, is by examining the head. But we believe, that in the features of the face, and more especially in the expression of the countenance, we shall often find indications of mental faculties, and of what is passing within. Education, however, habit, and the situation of life in which we happen to be placed, greatly modify our original dispositions and propensities, and must ever tend to perplex the phrenological and physiognomical enquirer. The same difficulties, indeed, attend the physiologist and pathologist. How often do we find functions of organs modified by peculiarity of constitution or habits of life—how seldom do we see the same disease run parallel in two individuals, however similarly situated?—These considerations should induce us to look with a charitable eye on the imperfections and even the errors of phrenology and physiognomy.

